# Floor Cleaners as Helper Pets: Projecting Assistive Robots’ Agency on Zoomorphic Affordances

**DOI:** 10.1007/s42979-023-01769-2

**Published:** 2023-04-29

**Authors:** Sophie Alice Grimme, Avgi Kollakidou, Christian Sønderskov Zarp, Eva Hornecker, Norbert Krüger, Philipp Graf, Emanuela Marchetti

**Affiliations:** 1 OFFIS e.V., Institut für Informatik, Escherweg 2, 26121 Oldenburg, Germany; 2 Bauhaus-Universität Weimar, Bauhausstraße 11, 99423 Weimar, Germany; 3 University of Southern Denmark, Campusvej 55, 5230 Odense M, Denmark; 4 Chemnitz Technical University, Straße der Nationen 62, 09111 Chemnitz, Germany

**Keywords:** Ageing adults, Care home, Dementia, Assistive robot, Field study, Focus groups, Playful design, Playful interaction

## Abstract

Care of ageing adults has become a dominant field of application for assistive robot technologies, promising support for ageing adults residing in care homes and staff, in dealing with practical routine tasks and providing social and emotional relieve. A time consuming and human intensive necessity is the maintenance of high hygiene quality in care homes. Robotic vacuum cleaners have been proven effective for doing the job elsewhere, but—in the context of care homes—are counterproductive for residents’ well-being and do not get accepted. This is because people with dementia manifest their agency in more implicit and emotional ways, while making sense of the world around them. Starting from these premises, we explored how a zoomorphic designed vacuum cleaner could better accommodate the sensemaking of people with dementia. Our design reconceptualises robotic vacuum cleaners as a cat-like robot, referring to a playful behaviour and appearance to communicate a non-threatening and familiar role model. Data from an observational study shows that residents responded positively to our prototype, as most of them engaged playfully with it as if it was a pet or a cat-like toy, for example luring it with gestures. Some residents simply ignored the robot, indicating that it was not perceived as frightening or annoying. The level of activity influenced reactions; residents ignored our prototype if busy with other occupations, which proves that it did not cause significant disturbance. We further report results from focus group sessions with formal and informal caregivers who discussed a video prototype of our robot. Caregivers encouraged us to enhance the animal like characteristics (in behaviour and materiality) even further to result in richer interactions and provoke haptic pleasure but also pointed out that residents should not mistake the robot for a real cat.

## Introduction

In 2018, around 9.1 million people over 60 were diagnosed with dementia in EU states [1]. Dementia describes a variety of brain disorders which progressively lead to brain damage, causing deterioration in memory, thinking, behaviour and the ability to perform everyday activities [1]. Many of these individuals can still live at home, but when reaching a later stage of the disease, most will eventually move to a residential care facility.

Dementia harms not only the individuals affected, but also caregivers and families, causing physical, psychological, social, and economic challenges [2]. Moreover, the number of people working in elderly care is decreasing. The job requires long working days and shifts, physically hard labour, often at relatively low wages and with too little time for caring for residents [3–5]. According to Riek [6], there is a substantial health-care shortage, because far more people need care than healthcare workers are available to provide. Therefore, researchers in healthcare robotics have proposed multiple robotic solutions for providing health support. Amongst other things, robots may help people with cognitive impairments, support caregivers, and aid the clinical workforce [6].

A common and time-consuming task in care homes and hospitals is cleaning the floors. In the care home where we conducted our study, a robotic vacuum cleaner had been trialled. The caregivers reported that the residents, most of whom are at later stages of dementia, were overwhelmed by the robot, because they could not perceive it easily due to its dark colour, and felt unsettled by its unpredictable movement pattern and noise level. Therefore, the cleaning robot could not be used any further.

**Fig. 1:**
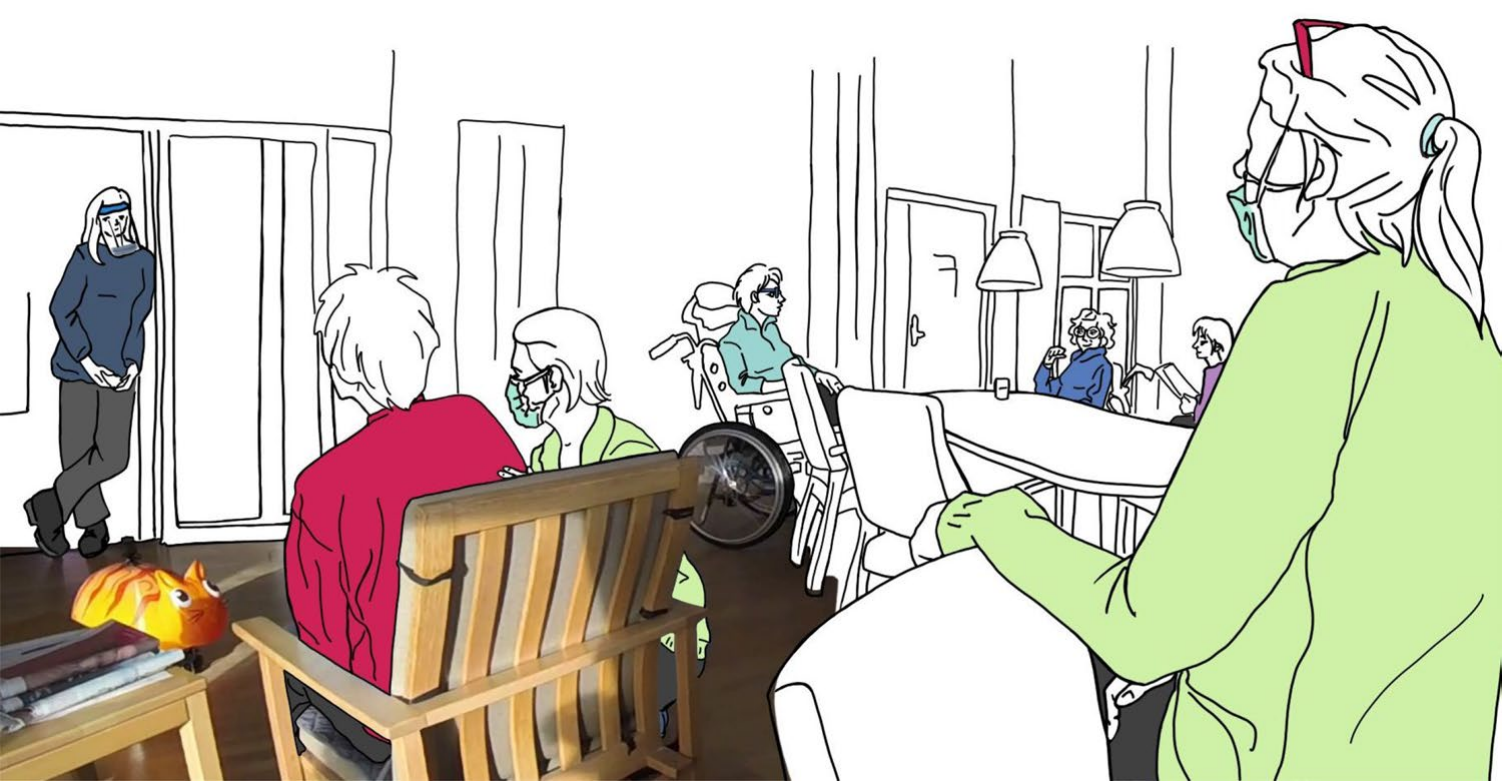
Our Sanne prototype moving across the shared room during tests. Left in the door, one researcher steers Sanne through the room. On the right, another researcher takes field notes

The project reported here focuses on developing a mobile cleaning unit with a playful design, to be used at care homes for people with dementia. We investigated the design of a robotic appliance, which has the functional purpose of cleaning the floors autonomously, but also a social purpose for residents. We aimed at a vacuum cleaner that does not elicit the feeling of being overwhelmed and may even lead to amusement. We designed our robot in the form of a toy cat, so that it could serve as a pet-like mascot (Fig. 1). Residents’ reactions to the robot were investigated through in-situ observations in two Danish care homes. The focus of our analysis was: *whether a playful, zoomorphic design for such a robot, with a playful moving pattern, will be accepted by people with dementia, without them getting scared or overwhelmed*. In our study, we define residents’ acceptance as a positive user experience, characterised by an open, possibly playful attitude towards our prototype [7]. Moreover, in parallel to the in-situ study, we conducted focus group sessions with people working in or involved with elderly care, to discuss the concept, design and possible pitfalls.

In the following Section “Background and Related Work”, the background of our study and related work are discussed, while the design process is presented in Section “Designing Nonthreatening Robotic Cleaners”. The user study and findings regarding the reaction of residents are described in Sections “The Study” and “Findings”. Section “Focus Group Feedback” describes the focus group sessions and the feedback collected. Hence Section “Discussion” proposes a critical discussion of our results and future work, and Section “Conclusion” the conclusion.

## Background and Related Work

The estimated number of cases of dementia will almost double by 2050, growing from 1.57 to 3.00% of the European population [8]. Studies show a positive effect on cognition through preventive interventions, a healthy diet, physical exercise, and cognitive training [2]. Nevertheless, there is no cure or disease-modifying treatment for dementia yet.

Although dementia mainly affects older people, it is not a normal part of ageing [2] and comes with different challenges for each patient. Dementia has different stages, characterised by different signs and symptoms. The late stage of the disease comes with behaviour changes and difficulties in recognition of humans and objects, causing an increased need for assisted self-care [2]. The participants of our study were almost all in this later stage, therefore their needs have to be considered when designing technology for care homes.

According to current literature [9], ageing adults affected by dementia experience changes in their perception of the environment and corresponding behaviour. This occurs in different ways, in some cases people may lose control of their emotional responses to environmental stimuli, leading to erratic behaviour and emotion, or show indifference to their surroundings [10].

In the study of dementia, it was found that laughter and humour can bring significant benefits to people affected [10]. Laughter has been acknowledged as a supportive method to complement clinical treatment, improving quality of life. Another important aspect for well-being is the use of familiar objects to sustain daily practices [9, 11]. Dementia causes cognitive impairments, which hinder people from understanding the function of new unfamiliar objects. Nevertheless, when interacting with own objects and perceptually similar ones, people are able to spontaneously relate to and use those objects in their daily practices [9], whereas perceptually unfamiliar objects were not easily understood by the same study participants [9]. It seems that using certain objects for many years, people internalise their physical use and context [9, 12], so that despite of dementia they can still actively relate to these. This can be explained as an effect of implicit memory, an unconscious memory generated by previous experience of repeated task performances, not linked to specific episodes [13]. Repeated exposure to objects results in perceptual priming that is resilient to cognitive impairment and can support people affected by dementia in performing their daily practices [13]. Thus, the design of novel technologies and artefacts for residents in care homes should take into account perceptual familiarity, so that novel artefacts fit with residents’ previous experiences and sociocultural context.

When designing a mobile robotic unit, we have to be careful to not trigger fear of falling [14]. Falling is acknowledged as “the second leading cause of death from unintentional injury” [14]. Dementia is known for leading to unconscious wandering, agitation, and perceptual difficulties, which in combination with the physical fragility associated with ageing might cause people to experience serious injuries [11]. As a consequence, ageing people affected by dementia, manifest a strong fear of falling [11, 14].

The design of assistive robots for care homes typically includes automated mobile units, which are in charge of carrying things around, monitoring safety, engaging people in physical exercise, even including exoskeletons and wearable devices aimed at improving physical mobility [6]. Thus, no matter whether care home residents will actively use such robots, or just encounter them in their daily environment, fear of injuries should be considered as a key factor in such designs for the residents’ safety.

Building on these insights, we aimed at designing a mobile floor cleaning unit, which could be perceived as nonthreatening, in relation to three main aspects: humour, familiarity of objects, fears of falling and injuries.

## Designing Nonthreatening Robotic Cleaners

The work presented here is part of a larger research project on care home technologies. In this context, interviews and (participant as well as non-participant) observations at care homes were conducted. The researchers also visited the cooperating two care homes of OK-Fonden in Odense, Denmark.^1^ Here, the inspiration and motivation for the reported work was gathered. This was complemented via a number of (online) interviews and meetings. When the design was finished, the pandemic situation allowed to test the prototype at the care homes of OK-Fonden.

### Development Process

This research followed the Human-Centred Design approach (closely related to User-Centered Design), which aims to create usable and useful systems and products by focusing on users, their needs, and requirements [15–17]. During the initial stage of the project, the setting, the care staff and a clinical clown, whose role is to activate and engage the residents, were observed. The goal was to gain a first-hand understanding of the user group and context of use, identifying relevant users and stakeholders [18]. This yielded insights into the daily life in the care home, the activities, behaviour and challenges for residents and staff. It revealed the omnipresent problem of keeping the floors clean and the mentioned problems when using a vacuum cleaning robot. This robotic vacuum cleaner was hard to perceive for residents due to its dark colour and minimalist round shape, which, according to the staff, made it look like a black hole moving across the floor. Moreover, its movement pattern was unpredictable for the residents, making them restless. Therefore, the acquired robotic vacuum cleaner was discarded after only a few months, because of its negative impact on the residents.

The clinical clown was a source of inspiration for investigating patterns of movement and appearance of our robotic vacuum cleaner. She dresses as a cow and uses a slow and predictable as well as playful movement pattern to approach people. This was reported as being especially important for residents with dementia, who are not frightened by such a slow and playful approach pattern.

After discussing requirements among the researchers, it was decided to design a playful interactive mobile cleaning unit. Given the positive effects documented of laughter and humour on people with dementia [10], we experimented with a playful, zoomorphic look for our prototype, to elicit positive emotion and laughter. The concept prototype is nicknamed “Sanne”, short for ’sanitizing unit’. As according to [18] interviews with management and staff members of the care home, the board of OK-Fonden and a hygiene expert of a hospital, provided feedback to this idea as well as additional insight into residents’ and stakeholders’ needs. Staff argue that residents are cognitively and emotionally affected by colours; red and orange are stimulating due to its vibrant chroma [19, 20], while white and black can be hard to perceive. On the other hand, blue and green were described as pleasant and relaxing, but easily ignored by residents. Therefore, it was suggested to colour our prototype red and orange to make it easily visible for residents. Potential shapes of the cleaning unit were also discussed. A staff member reported that the residents of this care home felt uncomfortable and ’unnatural’ while interacting with a robot toy resembling a seal, which sparked the idea to use the form of a domestic animal, which should be familiar to residents and can be expected of moving around (cp. [9, 11]). The size of the cleaning robot was also decided in collaboration with the staff. According to a hygiene expert, the floor itself is considered potentially infectious and unhygienic, and thus the body of the robot (especially the head that might be touched by residents) needs to be at least at 20–30 cm height, to avoid contamination. On the other hand, the robot should still be able to drive under furniture (Fig. 1).

During the design process, the stakeholders continued to contribute feedback and ideas. After the first digital sketches, use cases and user scenarios were created, also considering potential challenges. Hence, a video-prototype consisting of animated sketches was created and used as a stimulus in the focus group sessions, to gain additional feedback. Finally, the first prototype was created based on our understanding of the users, tasks, and environment [17].

As mentioned, the shape of a cat was chosen as a familiar pet. The robot cat was intentionally designed to look toy-like, to avoid deceiving the residents about its nature as an inanimate object. Orange was picked because of its activating nature, and being easy to perceive to reduce the risk of tripping.

### Proof of Concept Prototype

Our Sanne prototype was developed with the TurtleBot,^2^ a modular robotic unit widely used in teaching and research environments, due to its small size and flexible design. We selected the TurtleBot, mainly because it was within the desired size limits, price tag, and it can support a fast, modular prototyping process. The TurtleBot is equipped with a raspberry Pi, raspberry pi Camera, and 360$^{\circ }$ 2D LiDar among other sensors. These will be vital for further development, e.g. autonomous navigation in the care home environment.

**Fig. 2:**
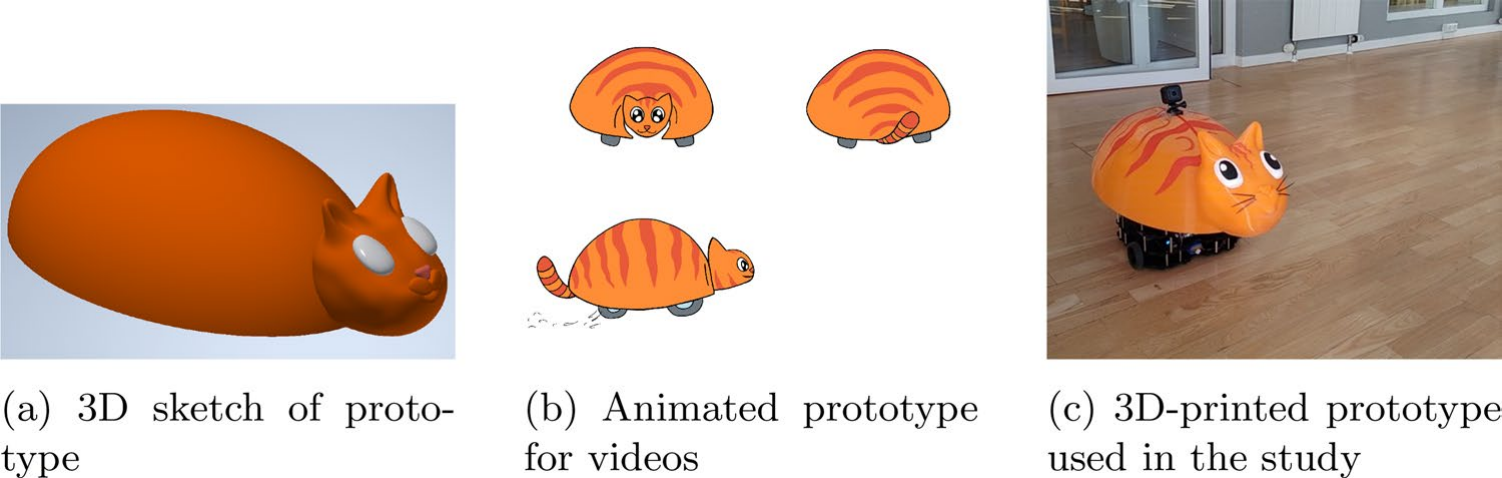
Different stages of development of the Sanne prototype

A 3D-printed cover with the design of an orange cat was mounted on top of the TurtleBot. This first prototype weighs around 4 kg, is 80 cm long from head to end, 40 cm wide and around 40 cm tall, so to be able to move under furniture, while avoiding contamination from the floor, as pointed by the hygiene expert. Moreover, the staff should be able to move it, if stuck in furniture and in case of malfunction. For research purposes, Sanne had a camera mounted behind her ears, so to record reactions of residents and staff (see Fig. 2).

Currently we are still experimenting with acceptable movement patterns, regarding speed and quality of movement when approaching residents, so to be seen, and to avoid scaring and intruding in residents’ activities. In the tests, Sanne slowed down when approaching residents and moved faster when further away. In addition, a ’wiggling’ movement was used when Sanne was close to residents, by driving back and forth and sideways, to make Sanne look playful and attract residents attention. This pattern was inspired by the clown’s movement.

For the study, we utilised a Wizard-of-Oz setup, which is a common method used for exploring how humans react to autonomously moving objects and robots before having fully functioning prototypes [21, 22]. This means that we remote-controlled the movement of Sanne, enabling us to flexibly react to emerging situations and adjust on-the-spot to the residents’ needs.

## The Study

The evaluation of our proof-of-concept prototype was conducted in the field, that is at our partner care home. Our study focused on the perception of Sanne, residents’ acceptance towards our robotic prototype, and on movement patterns suitable for a care home. We tested the mentioned movement pattern while observing the residents’ reactions in relation to their current activity level.

We conducted our test in two care homes of our partner institution OK-Fonden, in 6 different house units. In total, 30 residents (16 females, 14 males) participated in our study. Their age and names were not recorded, as for our study the state and effects of the dementia disease were more relevant than personal information. The process of the study was approved by OK-Fonden’s management. Informed consent was obtained from the resident’s relatives for their participation. For presentation here, participants have been anonymized via drawings based on the recorded videos.

Our tests took place on 3 days at different times and lasted 30 to 70 min. To investigate if and how the residents’ activity level might influence their perception of Sanne, we showed them our prototype before and during lunch, which qualify respectively as low and high activity levels. In total, 2:50 h video footage were gathered. One camera was mounted on top of the robot’s head, approximating Sanne’s field of view, to record close-ups of the residents. A second camera was installed in the room, to record the space and document other eventual interactions between the residents and how Sanne might affect these.

As the researchers did not speak Danish fluently, a staff member of the care home was present to help communicating with the residents and intervene in any unexpected situation. The researchers wore a care staff uniform, to blend in and not raise too much attention. They also had to wear a face shield and keep distance from the residents, and were tested negative, due to hygienic restrictions related to the COVID-19 pandemic as shown in Fig. 1.

One researcher controlled the robot via a remote control, according to the Wizard-of-Oz method, to simulate that the robot was moving autonomously. This researcher stayed in the background and tried to hide the remote control, to avoid that the residents might notice it. A second researcher stayed close to the residents, to take notes of their reactions and their words (Fig. 1). Attending care staff were sitting or standing next to the residents, talking to them or supporting them in daily activities.

Later, we conducted a series of follow-up semi-structured interviews, the first with the staff of the care home and the second one with its management. Both interviews lasted 30 min and were recorded. These interviews focused on the tests and staff’s regular daily practices and challenges. The aim of these interviews was to gain an understanding on whether the caregivers noticed any unusual moods or reactions, caused by Sanne’s presence in the home. Since Sanne is supposed to ease the daily work of the care staff in keeping the home clean, we asked them how they perceived Sanne, to comment on her potential and which difficulties they could foresee. The interview with care home management focused on the general concept of Sanne and how the user tests were experienced.

As mentioned, feedback on the concept of Sanne was collected in online focus groups with German caregivers using an animated video-prototype. These participants were informal and professional caregivers, recruited via newspaper ads in local newspapers, flyers and word of mouth. Responses and suggestions from these sessions are reported after the in-situ study findings.

## Findings

The recorded video footage provided the key data for our analysis. The data was overseen in total and transcribed into a logbook containing meta data but also a first description of the situations and contexts recorded. Spoken audio was translated and added to the document. The whole material was then labelled to identify significant occurrences that can be used in a comparative manner, such as: residents’ reactions to Sanne, their activity level, and how they became aware of Sanne during the test. In total, 37 situations were identified for a further and deeper analysis. We focused especially on occurrences in which the residents noticed Sanne and reacted with a behaviour that can be interpreted as specific and social interactions with it. Some situations were then sequentially reconstructed shedding light into situational interpretations of the residents interacting with Sanne.

**Fig. 3:**
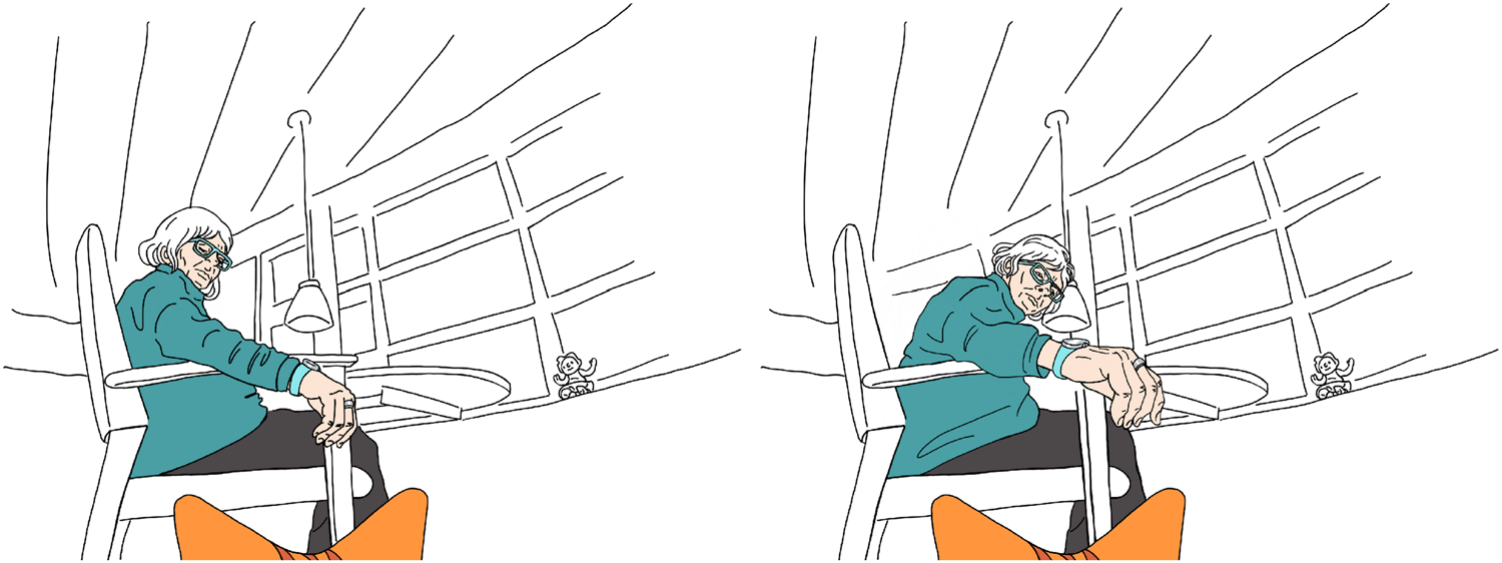
Positive reaction, luring with hand

### Reactions to Sanne

The residents’ reactions were categorised into positive, neutral, negative, and none-reaction. *Positive reactions* towards the robot include situations in which the residents showed interest in Sanne by asking questions, talking about or to her, smiling at her and luring Sanne to come closer or touching her (Fig. 3). Situations where residents only noticed and visibly accepted her presence or commented briefly, but did not further interact or relate to Sanne, were categorised as *neutral reactions*. Negative verbal comments, a perceptibly worsening mood or a rejecting gesture were categorised as *negative reactions*. Situations, in which residents could not perceive Sanne because of their activity level or health condition, as well as situations where they did not show any interest, ignoring Sanne, were labelled as *none-reaction* (both types are counted as none-reaction as it is not always possible to tell whether Sanne was intentionally ignored or not visible from the point of view of the resident).

16 out of 37 reactions were positive, only one was negative. 11 residents reacted neutral, and in 9 situations, no reaction was discernible (Table 1).

**Fig. 4:**
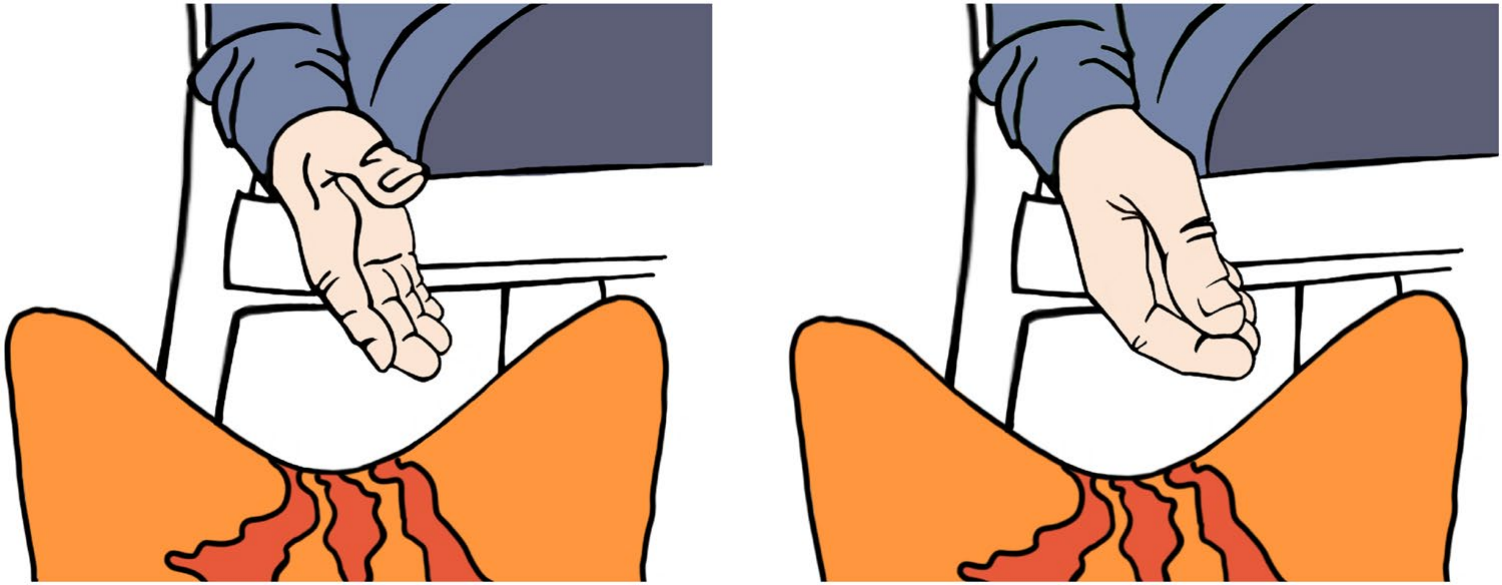
Close-up of luring gesture with hand

The four reaction types were subcategorised according to the kind of reaction (Table 1). Residents who reacted positively, were either talking to or about Sanne, luring and/or touching her. In three situations, Sanne was touched by residents, they either scratched her head or touched her ears. Before every touching situation, the participants talked to Sanne or lured her. While talking to her, some participants altered their voice to reach a higher tone, reminding of the tone used while talking to children or pets. Luring the robot was done either by whistling, reaching with the feet towards Sanne, or via a hand luring gesture (Fig. 4).

**Table 1:** Occurrences of reactions to Sanne and sub-types of reactions

Reaction	Situations	Sub-type	Situations
Positive	16	Talking	5
		Talking and luring	5
		Luring	3
		Talking, luring and touching	2
		Talking and touching	1
Neutral	11	Observing	7
		Listening and agreeing	3
		Listening	1
Negative	1	Kicking	1
No reaction	9	Not interested	5
		Not possible	4

Residents who showed neutral reactions either just observed Sanne or listened to a caregiver, or employee talking about her and agreed to what was said. In three of the 11 neutral situations the conversation went about like this:

- *Caregiver: Look, the cat is coming over there.*
- *Resident: What is it doing?*
- *Caregiver: It’s supposed to wash our floors, wouldn’t that be nice?*
- *Resident: Yes, of course!*

**Fig. 5:**
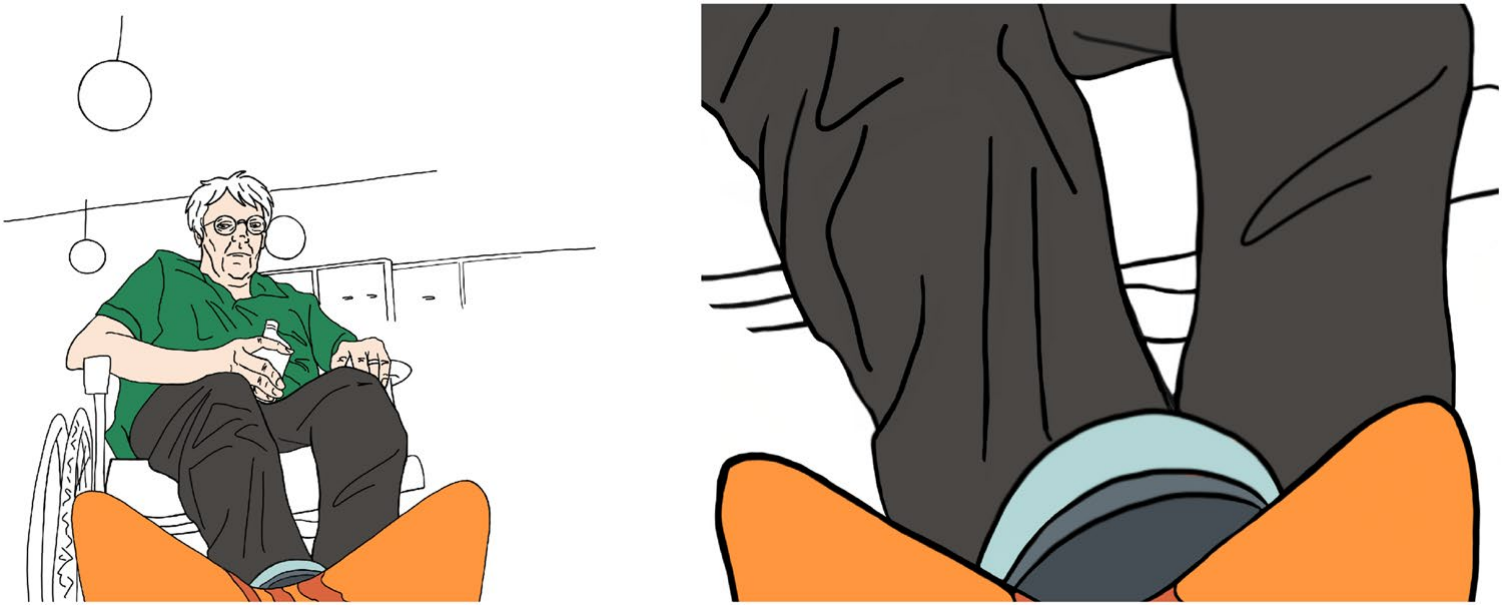
Negative reaction, kicking at Sanne

The one negative situation observed was labelled as such because the resident followed Sanne and kicked the robot (Fig. 5). Nevertheless this situation was ambiguous, as he did not show visible rejection, fear or anger and continued to follow Sanne. The resident appeared negative concerning our study in general, as he also kicked a researcher to clear his way. Kicking is usually done to show dislike or to keep something at distance, and was therefore categorised as negative.

In some cases, no reaction was observed, this happened typically when residents were focused on other activities and did not notice Sanne. Some seemed to see her, but did not appear interested, so that it was not possible to determine if they would accept her or not.

### When did Residents Take Notice of Sanne

**Fig. 6:**
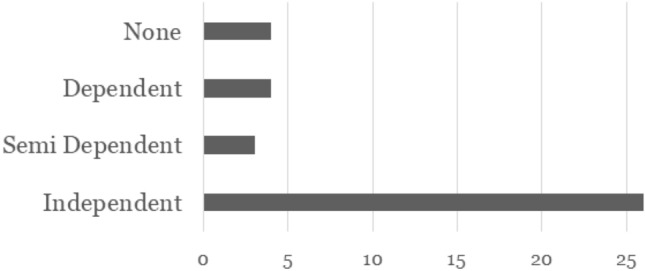
Degree of ability to notice Sanne

As another key finding, we consider how the residents became aware of Sanne’s presence. During the user study, at least one caregiver or employee of the care home was always present, which led to conversations about Sanne. To determine whether Sanne will be able to drive through the care home by herself without needing somebody to introduce her or to make the residents aware of her, we analysed how many people were able to perceive her without any help. There are four different categories of how easily residents could take notice of Sanne (Fig. 6).

From 37 situations, 26 residents were able to notice and reacted to Sanne *independently*. In those, no help was needed from a caregiver, employee, or other resident to perceive the robot and to react. *Semi-dependent* were residents who either needed help to shift their attention towards Sanne or to react. Only three residents needed this partial help.

1. *Caregiver: Look who is coming here!*
  *Resident: Oh yes, is it walking on wheels?*
2. *(Resident observing Sanne on the floor)*
  *Caregiver: Do you think its cute?*
  *Resident: Yes.*

The category *Dependent* summarises the situations where residents needed help to notice Sanne and react to her. The caregiver or employee lead the conversation about the robot, and it is not clear whether the resident would have noticed Sanne without any help. Situations like this were observed four times.

- *Caregiver: Look there! Resident: Ah.*
- *Caregiver: What do you think about it, is it nice? Resident: Yes.*

The last category describes situations, in which the residents did not pay any attention towards Sanne, in spite of the effort of a caregiver or other residents. This lack of attention was observed four times and assigned to the reaction category *None* and subcategory “Not possible”.

To provide more insight into how residents encountered Sanne, we here show one detailed example. Resident Maxine sits at a table with other residents. Sanne tries to find a way around the chairs and wheel chairs (remote-controlled by our Wizard-of-Oz operator) and finally approaches Maxine.

- *A staff member asks Maxine to stand up to see what’s coming on the floor. Once Maxine detects Sanne she smiles.*
- *Maxine: “Nooo! Where does that come from?”*
- *Staff: “I don’t know. It’s here to visit.”*
- *Maxine: “Oh it’s really cute, where is it from?”*
- *(Staff points into researchers direction.) *
- *Staff: “Those guys over there, they are here to visit us.*
- *(Maxine points to researchers)*
- *Maxine: “Oh it’s really cute.”*
- *Staff: “I think so too”*
- *(Staff bends over to Sanne)*
- *Maxine: “What’s it’s name?”*
- *Researcher: “Sanne.”*
- *Maxine:“Can I touch it?”*
- *Staff: “Yes.” *
- *Researcher: “It’s a toy that’s supposed to wash the floor and we’re hoping it could be a fun thing.” *
- *(Maxine touches Sanne at the nose with the back of her fingers, looks into the eyes and then into the camera) *
- *Maxine: “That’s fantastic!” *
- *Staff: “And it can come and visit you some times.”*
- *Maxine: “Oh, that’s nice!”*
- *(Maxine sits down and turns around to another resident next to her and points at Sanne.)*
- *Maxine: “Have you seen it?”*
- *(The situation lasts for another minute where the Staff and Maxine try (but fail) to direct the attention of the other resident to Sanne.*

This example shows how a staff member asks the resident if she wants to get up to look at something, but doesn’t reveal what this may be. This leads to an authentic first reaction of the resident. The “No” at the beginning of Maxine’s first statement is similar to a friendly exclamation when one sees something very cute or beautiful. Maxine shows interest by asking about the name and origin of Sanne. The staff member tells Maxine that Sanne will be cleaning and she responds: “That’s fantastic!”, indicating her acceptance for Sanne’s intended future function.

### Influence of Activity Level

**Fig. 7:**
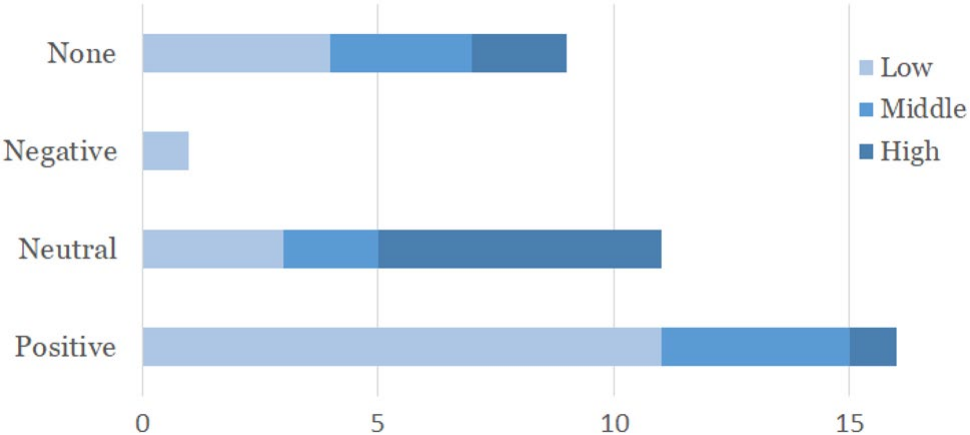
Influence of activity levels on reaction types

The third aspect of our analysis deals with whether the activity level of the residents might have influenced their reaction to Sanne. Activity levels were categorised in low, middle, and high (Fig. 7).

A *high activity level* is represented by situations in which residents are busy during a meal, talking to somebody, or focused on an activity such as handicraft. Instances in which residents were watching TV, reading a magazine, walking through the care home, or having a small snack or drink, were categorised as a *middle activity level*, as these did not require intense focus from the residents. Situations where people were only sitting or standing somewhere were categorised as *low level activity*.

The prototype was tested before lunch (low to middle activity level) and during lunch (high activity level). From 16 positive reactions to Sanne, only one resident had a high activity level, while 11 residents had a low level. Six of the 11 neutral reactions happened during high activity levels. In general most (19 of 37) situations were observed during the residents had a low activity level (Fig. 7), including also the largest number of positive reactions. That Sanne is largely ignored during high activity levels indicates that the robot does not distract residents (e.g. during lunch). That most positive reactions occurred during low activity levels is encouraging regarding our design intention to bring some light-hearted humour into the daily life of the residency home.

## Focus Group Feedback

In parallel to preparing the in-situ study, as part of the larger project that the development of Sanne is part of, focus group sessions were run by the German project team. These sessions were originally planned to take place face-to-face and before building Sanne, but the Corona pandemic necessitated re-planning and increased the level of preparation needed. Therefore, the feedback received could not be taken into account for the current design of Sanne. Nevertheless, we take some recommendations and ideas from these for the further development of our playful friendly cleaning robot, and gain additional insight into the perspectives of caregivers and people involved in the larger context of elderly care.

We recruited people in Germany who work in elderly care either as professional caregivers, in related areas, or as informal caregivers for relatives. Recruitment was done via advertisements in local newspapers and social networks, via care home workers and managers that the project had been in contact with, and via snowballing—with the local newspaper being the most effective method. Groups of three to four participants each discussed three to five concepts for care robots (all concepts had been developed within our project ReThiCare) in an online session of 2–2 1/2 h. How many concepts could be discussed depended on how long discussion took in each group. Prior to the sessions, participants received a flyer with information on the project, a consent sheet and a notebook with short descriptions of the concepts and space for written notes. This notebook was to be used before and after the main discussion and then to be sent back to us by postal mail. We made sure all concepts were discussed about the same number of times by all status groups; the order in which concepts were discussed was also randomised. Sanne was discussed by eight focus groups, in total with 19 people. The majority of participants were professionals regarding the care context.

**Fig. 8:**
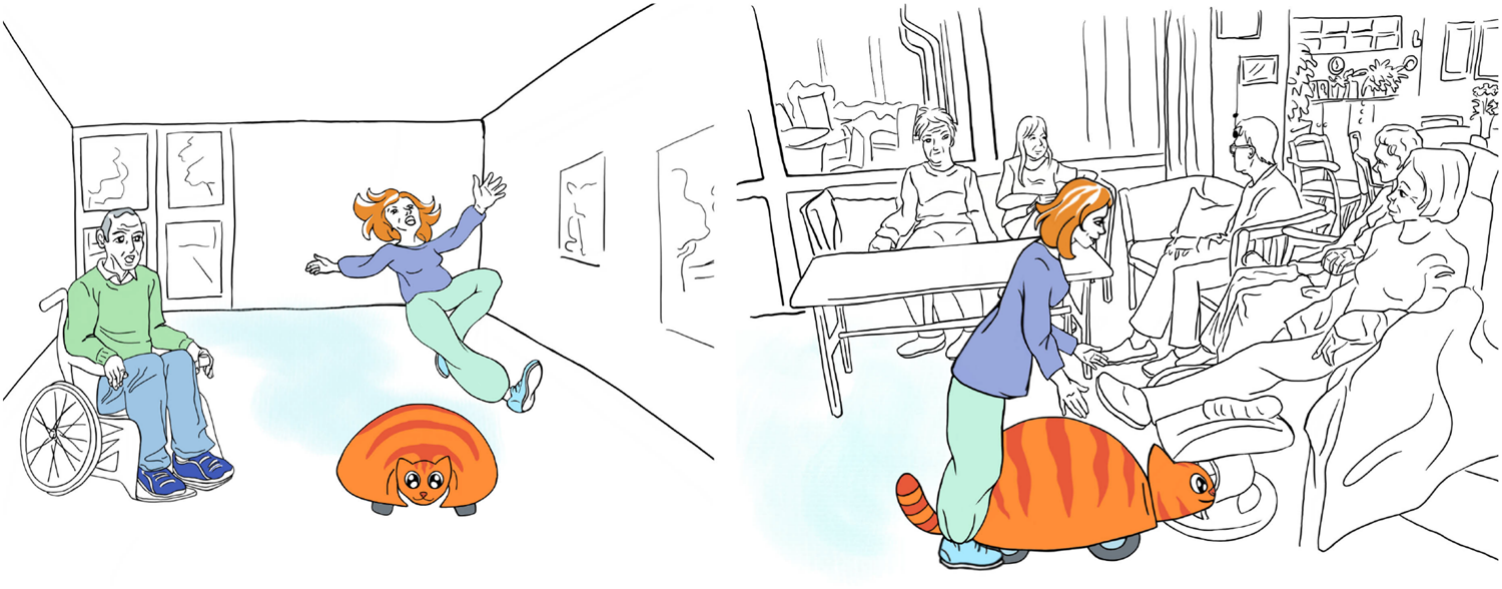
Two stills from video used to ground discussions, showing potential negative scenarios: (Left): Woman slipping on a wet floor after Sanne has washed the floor. (Right): A caregiver has to move Sanne after it got stuck under a footrest

As stimulus for the discussion, video-prototypes had been developed to show the concept in the context of care work. The animated video of Sanne showed several scenarios of the robot in a nursing home: Sanne cleaning in a living space while a resident lures and pets the robot. Another scenario shows Sanne moving near a crowded dinner table. Finally, one scenario illustrated potential problems in the interaction with the robot, such as someone tripping (see Fig. 8). The latter ’negative’ scenarios (inspired by the notion of ’contravision’ [23]) were included purposely to encourage critical discussion and to explicitly invite such feedback.

In addition to these group sessions, we conducted a number of expert interviews with individual caregivers or managers that had knowledge on specific topics such as dedicated therapy forms for people with dementia or the organisational context of care homes, to gain insight into specific needs and requirements.

All sessions were recorded (with participants’ permission), and subsequently analysed. For each session, two project team members acted as facilitators for the discussion, while a student assistant supported them by taking notes and managing the technical aspects of the online sessions, for example, taking care of screening the video-prototype. To ensure accessibility to our discussion regarding technical accessibility, we provided participants the choice between several technical platforms, such as Zoom, Skype and BigBlueButton, and then allocated them into groups that had opted for the same platform. In two cases we also conducted telephone interviews with a few participants, in particular older people, who lacked the technical equipment—microphone, speakers and camera—or general access to online conferences.

By testing the setup and procedure beforehand, we could resolve several problems. The most challenging issue was to ensure every participant was provided with the exact same stimulus of the video-prototype, which required external software and captions in the videos in case there were issues with the audio transmission.

**Fig. 9:**
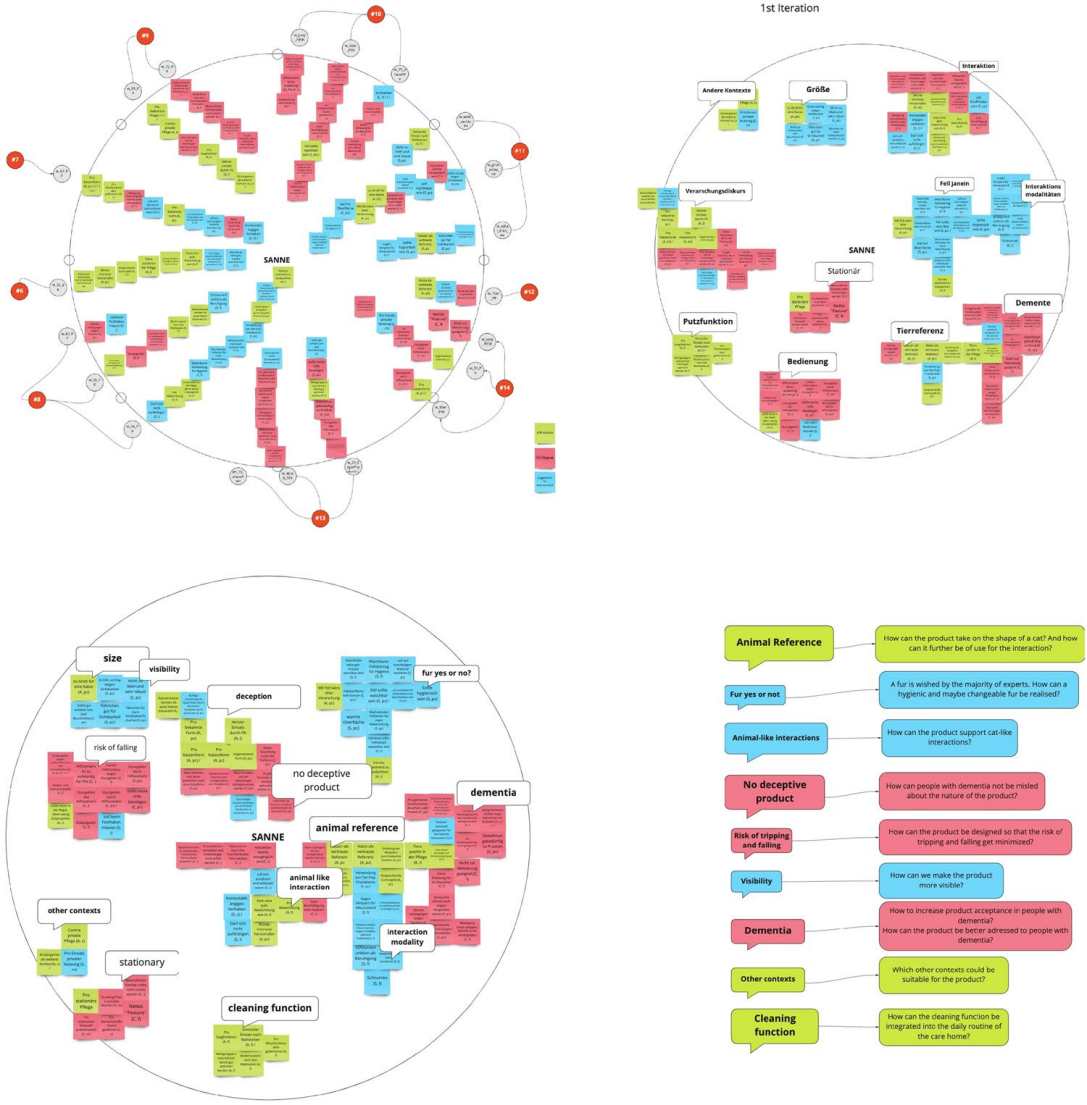
Overview over the process of the affinity coding process, from left to right, where green post-its represent positive, affirmative statements, red represents critical statements and blue for new (re-)design ideas that came up. We further labelled post-its regarding whether the statement concerned product characteristics, functionality, modalities, context of use, or interaction

We analysed the data using an extended approach of affinity coding. Affinity coding works on a visual level, enabling all team members to look directly at the analysed data. In the first step, we summarised opinions and evaluations given by our participants into short sentences. Then, we arranged all statements about one concept into a star/circle diagram, where each line lists the statements from one participant in rough chronological order—from the outside to the middle (see Fig. 9 left). In the next steps, we iteratively grouped the statements into semantic clusters and also visualised connections and relations between clusters. This enabled us to subsume different statements into various clusters regarding the idea. Finally, the clusters were translated into short and concrete questions (e.g.“How can a hygienic and maybe changeable fur be realised?” and “How can people with dementia not be misled about the nature of the product?”), which were provided to the entire project team for the further development process.

Participants confirmed that a cleaning robot that can be integrated into daily routines is desirable, in particular for vacuuming. They thought that having the robot wiping floors might be more dangerous, as freshly wiped areas would have to be cordoned off to prevent residents from slipping on the wet surfaces.

Almost all focus group participants agreed with the idea of using a cat as a familiar animal reference that elderly people may be able to relate to. They may have a calming effect. Also, given it is a pet, most people may be able to relate to, residents already know how to behave towards them. Many participants suggested that the robot could be enhanced with more cat-like behaviours and interactions, such as a moving tail or ears, fluttering eyes, and purring noises, which could engage residents more, provide entertainment, and result in longer interactions. The underlying argument was that it would be a positive experience for residents if Sanne responded to their approach like a cat, in a coherent, cat-like manner. This would fulfil expectations resulting of the role model of a cat and situational associations with it.

To indicate the direction of movement to the residents, a moving head or tail were originally considered. This will be investigated in further tests and design iterations, also with regard to movable ears. The suggestion to integrate sound reactions is something that we aim to explore in the future. Some participants further suggested to cover motor sounds with cat-like noises. They also recommended that Sanne should move slowly, as fast movements can be scary for people with dementia.

Whilst some participants remarked that elderly people should not be misled or deceived about the artificial nature of the robot, most encouraged us to conduct real life tests to answer this question. They also told us about residents they had encountered who maintained a proto-social relationship to a dementia cat robot, while at the same time being well-aware that the object was not really alive. The professional caregivers also anticipated that residents might throw food at Sanne to feed her, if they think she is a real cat, which would defeat the purpose of a cleaning robot. We therefore decided to further expand Sanne’s capabilities to act according to its social role on a behaviour level, while refraining from making shape and appearance too lifelike.

Many participants suggested that it would be good if Sanne had a soft surface or sort of fur. This would need to be washable and utilise hygienic, anti-bacterial materials that can be disinfected, to be practical within the daily routines of a care home. Feeling fur could provide pleasurable haptic reward when stroking Sanne and thereby meet the need for haptic stimulation, which is often experienced by people with dementia. Yet fur could negatively influence whether Sanne is easily identifiable as a machine for people with dementia. Thus, this suggestion requires further discussion, careful design and more user studies.

While focus group participants liked the animal-reference and the overall idea of Sanne, they also voiced concerns whether people with dementia might get confused about the two functions combined in one object, and whether they might be scared of a mobile robot. As our study revealed, the latter was not the case—none of the residents reacted scared. Residents even at advanced stages of dementia either just ignored Sanne or reacted positively and interested. Nevertheless, comments about the potential noise produced by the robot and the importance for slow, careful movements will inform our further work. Given the current version of Sanne does not yet have a cleaning mode, it remains open for future studies whether residents would be confused about Sanne having two modes, one where the robot cleans floors and does not seek attention, and another where it shows the friendly attention-seeking behavior from the study described here.

Further, participants stressed that Sanne should not be pushy in seeking attention, as this could annoy residents. As our study revealed, the behaviour pattern that we had planned fulfilled this requirement, as some residents clearly preferred to ignore Sanne (without signs of annoyance), or were busy with other activities, while being able to continue focusing on this activity despite Sanne’s presence in the vicinity.

Focus group participants further confirmed the concerns expressed by some of the staff in our Danish care home partner, that the risk of tripping over Sanne should be further reduced by making her even more visible and larger than shown in the video scenario. Note that the video that the groups saw did include tripping as a potential problem, and this thus was feedback explicitly asked for. While some noted that people with dementia tend to walk around a lot (they have an increased impulse for movement), others noted that most of the time, residents in care homes would be sitting around, which might reduce the likelihood of someone tripping. Several participants noted that Sanne should not require assistance from residents, as this could increase the risk of falls.

Overall, the results of the focus group discussion showed that caregivers considered the Sanne concept to be worth pursuing and realistic in its ambition. Issues that had already been identified, such as the risk of falls, but also the difficult question about the right degree of realism for Sanne can be worked on further. In addition, we were able to collect suggestions for concrete design, which we want to incorporate in our follow-up work.

Compared to the feedback we received during the development of Sanne from the Danish care home staff and management, who were generally very enthusiastic about our project, German caregivers in our focus group sessions appeared to be more skeptical. Part of the reason for this might lie in the different organisation and funding of the care sector in these two countries, where in Denmark quite a lot of technology is already used in everyday contexts of care, especially regarding people with dementia. Moreover, the German participants reacted to a hypothetical scenario provided in a comic-strip type video, which might prompt more speculative discussion focusing on possible risks.

## Discussion

In human-robot interaction for care robots, anthropomorphic and zoomorphic forms tend to be reserved to robots with an intentional social function, that are to provide emotional support, such as Paro [24], whereas robots that target practical tasks (such as vacuum cleaning) have an abstract appearance, with a simple, minimalistic look. With our robot Sanne, we explored how a cleaning robot with a playful zoomorphic form and playful behaviour could integrate into the daily life in a care home residency, where previously, attempts to utilise a Roomba robot failed since it had scared residents.

Caregivers in our focus groups had little ethical concerns about Sanne as a cat, but were concerned that people with dementia might get confused by the double function of such “a cleaning cat”, or get scared. A key finding from our study is that **73% of observed residents appeared to accept Sanne**, which means that at its current state, our prototype was reacted to positively or tolerated by most residents. Only one negative situation was observed, and 24% of observed situations had no discernible reaction to the robot (none-reaction). These cases of no reaction were considered a positive finding, since the residents did not appear scared or annoyed, but simply went on with their activities, ignoring Sanne.

The observed negative situation was, as reported by the caregivers, explainable with the fact that the resident never liked animals in his life. Moreover, by previously kicking one of the researchers, this resident indicated to be annoyed by anyone or anything standing in his way. Even though some residents might not like cats or the chosen colour for Sanne, we can argue that our prototype did not cause any fear, disturbance, or anxiety. Since Sanne is primarily intended to clean the floor, and only secondarily to provide an opportunity to interact or play with her, the residents do not necessarily have to respond and should be able to ignore her. Our research question *“Can a playful robotic vacuum cleaner, like Sanne, be accepted by people with dementia?”* can, therefore, be answered positively at this current state of development.

Another key finding is that **Sanne is perceived as cat-like**, indicated by 11 out of 16 positive reactions ending with touching her. The behaviour of luring and touching could be counted an implicit behaviour, comparable to playful interaction with pets. Nonetheless, the residents clearly appeared to be aware that Sanne is not a real living cat, as some commented on her body being hard and even knocked on her head, showing that the design of Sanne evokes behaviours that allude to cats as well as toy pets, without misleading people affected by dementia.

**70% of the residents were able to react to Sanne independently.** This shows that the robot could be used in an everyday situation, moving around the care home without needing help from caregiver to be introduced or be supervised during interactions. In this way, our prototype should be able to fulfil the purpose of keeping the floors clean and relieving care staff of this task, as the residents could perceive, accept, and some even liked to interact with Sanne, opposite to what happened with the previous commercial robotic vacuum cleaner.

**58% of residents with a low activity level reacted positively to Sanne.** The most significant reactions to Sanne occurred during low activity levels. When residents were focusing on a task and thus had a higher activity level, they only reacted neutral or very briefly to the robot. This shows that people can easily ignore Sanne, which might fit the purpose of cleaning the floors without causing unnecessary distraction. Finally this finding can provide clues for caregivers to find a suitable time to use the robot for actively cleaning the floor (for example during lunch or another activity) and for using it as an entertainment and talking point for residents.

A limitation of our study is that residents only encountered Sanne over a relatively short time period. Novelty thus may be a factor contributing to the positive responses observed, however due to the severe levels of dementia of residents, this is likely to far less influence long-term engagement than in other contexts. Nevertheless, our study took place directly after a pandemic lockdown ended, so residents might have been extraordinarily happy about anything that distracted their daily routines. Furthermore, the current prototype of Sanne does not produce the noises that a cleaning robot would emanate.

We have further gained insights from the focus groups for further development of the concept for our cat-like cleaning robot but also for pet-like devices in general. Most important here is to keep the right balance between designing an abstract figure of key characteristics and a more realistic appearance. While on the one hand, it is important to make such an object recognisable as a specific pet—so people can attribute a concrete mental model to it and act accordingly—on the other hand, it is ethically mandatory that people cannot be deceived about its artificial nature. In the case of Sanne we solved this tension by deciding to implement behavioural elements of our role model while at the same time keeping a comic-like appearance that is practical in this context, as it is easy to clean and to perceive. This also seems to be easier then the other way around, concretely designing a cat-like appearance but giving it a deviant or robotic-like behaviour, since this would be at risk of fulfilling pre-conditions of the so called uncanny valley phenomenon.

### Future Work

According to our study, Sanne might become a source of danger to those residents who have difficulties to attend to more than one thing at a time, or to anything that is not in their line of sight, a known issue for late stages of dementia [14]. In one instance, a resident was walking up and down the hallway and did not notice the robot on the floor; if the researchers would not have controlled the robot, a tripping incident might have occurred. Therefore, further investigation is required to develop a safety protocol for Sanne, fine tuning her movement pattern, establishing a safe distance and acceptable warning signals for residents in transit.

The observed negative situation revealed that Sanne should be able to react to residents’ rejections. Actions like kicking or utterances like *“Go away”* can be interpreted as warning signals, which Sanne should be able to detect and to shut off or drive away in response, avoiding further annoyance.

At the current stage of our research, it is not possible to make any claims about the noise level emitted by Sanne, as the tested prototype did not yet have a vacuum cleaning function. According to experiences reported by the caregivers of the previous cleaning robot and to concerns from focus group participants, loud noise can negatively affect the residents. Therefore, it is our plan to further investigate this aspect during in-situ workshops.

As the residents touched Sanne in 69% of the positive reactions, the tactile properties of the shell material should be further explored. Currently Sanne is made of 3-D printed plastic, which is not particularly pleasant, hence we aim to find a soft-feeling material, which invites touch but is also hygienic, so that it does not summon more work from the care staff for disinfecting the robot. As suggested in the focus groups a soft and pleasant material should be chosen, but it should not give the impression of touching a living cat, so that residents are not deceived about Sanne’s mechanic character.

## Conclusion

In our study, we explored the design of a non-threatening robotic vacuum cleaner for care homes, addressing in particular the needs of people affected by late stages of dementia. Our study was conducted through a Human Centred Design methodology. Therefore, we engaged in a close partnership with two care homes located in Odense, Denmark.

Our study demonstrates that people at late stages of dementia can accept a robotic vacuum cleaner, if it carries playful qualities in its appearance and movement pattern. Based on our conversations with staff, we gave our prototype, which we call Sanne, the appearance of an orange red-striped cat and a movement pattern, which supports visibility and predictability, avoiding fright and tripping.

Our results show that residents responded positively to our prototype, as most of them tried to engage with our prototype to play as if it was a pet or a cat-like toy. Only one negative response was observed, which was a rejection, but not accompanied by fright or accidents. Other residents simply ignored Sanne, as she was not in their field of view or they were engaged in activities. We consider non-responses as positive, as people are entitled to interact with Sanne or not according to their wish. These occurrences mean that there might be a risk of tripping, therefore, our next step in development will focus on exploring a safety protocol to avoid accidents while Sanne is cleaning the floor or simply sharing space with residents.

## Footnotes

1 https://ok-fonden.dk/.

2 https://www.robotis.us/turtlebot-3-waffle-pi/.
